# Serotonin (5‐HT)_2A/2C_ receptor agonist 2,5‐dimethoxy‐4‐iodophenyl‐2‐aminopropane hydrochloride improves detrusor sphincter dyssynergia by inhibiting L‐type voltage‐gated calcium channels in spinal cord injured rats

**DOI:** 10.1111/cns.14890

**Published:** 2024-08-04

**Authors:** Rong Lv, Mingzhuo Li, Xun Chen, Shengtian Li, Nailong Cao, Baojun Gu

**Affiliations:** ^1^ Department of Urology Shanghai Sixth People's Hospital Affiliated to Shanghai Jiao Tong University School of Medicine Shanghai China; ^2^ Bio‐X Institutes, Key laboratory for the Genetics of Developmental and Neuropsychiatric Disorders (Ministry of Education), Shanghai Key Laboratory of Psychotic Disorders, Brain Science and Technology Research Center Shanghai Jiao Tong University Shanghai China

**Keywords:** 5‐HT2A/2C receptor, detrusor sphincter dyssynergia, L‐type voltage‐gated calcium channel, spinal cord injury

## Abstract

**Aims:**

To explore the role of voltage‐gated calcium channels (VGCC) in 5‐HT_2A/2C_ receptor agonist 2,5‐dimethoxy‐4‐iodophenyl‐2‐aminopropane hydrochloride's improvement of spinal cord injury (SCI) induced detrusor sphincter dyssynergia and the expressions of the 5‐hydroxy tryptamine (5‐HT) 2A receptors and VGCCs in lumbosacral cord after SCI.

**Methods:**

Female Sprague–Dawley rats were randomized into normal control group and SCI group (*N* = 15 each). Cystometrogram (CMG), simultaneous CMG, and external urethral sphincter electromyography (EUS‐EMG) were conducted in all groups under urethane anesthesia. Drugs were administered intrathecally during CMG and EUS‐EMG. Rats were euthanized and L6‐S1 spinal cord were acquired for immunofluorescence.

**Results:**

In SCI rats, intrathecal administration of 2,5‐dimethoxy‐4‐iodophenyl‐2‐aminopropane hydrochloride or L‐type VGCC blocker, nifedipine, could significantly increase voiding volume, voiding efficiency, and the number of high‐frequency oscillations. They could also prolong EUS bursting activity duration on EUS‐EMG. Moreover, the effect of 2,5‐dimethoxy‐4‐iodophenyl‐2‐aminopropane hydrochloride can be eliminated with the combined administration of L‐type VGCC agonist, (±)‐Bay K 8644. No significant differences were observed in CMG after intrathecal administration of T‐type VGCC blocker TTA‐P2. Additionally, immunofluorescence of the lumbosacral cord in control and SCI rats showed that the 5‐HT_2A_ receptor and Cav1.2 immunolabeling‐positive neurons in the anterior horn of the lumbosacral cord were increased in SCI rats.

**Conclusions:**

Our study demonstrated that 5‐HT_2A/2C_ agonist 2,5‐dimethoxy‐4‐iodophenyl‐2‐aminopropane hydrochloride may improve SCI‐induced DSD by inhibiting the L‐type voltage‐gated calcium channel in lumbosacral cord motoneurons.

## Graphical abstract

L‐type voltage‐gated calcium channel blocker, nifedipine, could improve spinal cord injury‐induced detrusor sphincter dyssynergia. 2,5‐dimethoxy‐4‐iodophenyl‐2‐aminopropane hydrochloride may improve SCI‐induced DSD by inhibiting the L‐type voltage‐gated calcium channel in lumbosacral cord motoneurons.

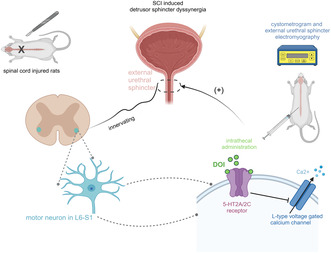

## 1 INTRODUCTION

The normal storage and voiding function of bladder detrusor and external urethral sphincter (EUS) are regulated by the central and peripheral nervous system. Unlike humans and other rodents, rats exhibit a distinct bursting activity of EUS during voiding phase with a frequency between 4 and 8 Hz.^1^ Spinal cord injury (SCI) can impair the normal neuronal control and can cause lower urinary tract dysfunction (LUTD). During the early stage, SCI causes hyporeactive bladder and urinary retention, while SCI‐induced LUTD is mainly characterized by detrusor sphincter dyssynergia (DSD) and neurogenic detrusor overactivity (NDO) in the late stage. DSD is defined as the impaired coordination between the bladder and EUS during voiding due to a neurological abnormality.^2^ Currently, the treatment for SCI‐induced DSD is limited, with anticholinergic drugs and botulinum neurotoxin mainly targeting detrusor overactivity as well as offering only mediocre efficacy.^3^ Interestingly, our previous studies demonstrated that intravenous and intrathecal administration of serotonin (5‐HT)_2A/2C_ receptor agonist 2,5‐dimethoxy‐4‐iodophenyl‐2‐aminopropane hydrochloride could enhance EUS bursting activity and restore the coordinated relaxation of the EUS in SCI rats.^4^ However, the mechanism of 2,5‐dimethoxy‐4‐iodophenyl‐2‐aminopropane hydrochloride for improving SCI‐induced DSD remains unknown.

Previous researches have demonstrated that the plateau potential of spinal motoneurons is involved in the pathophysiology of spasms or tonic activity of the muscles after SCI.^5^, ^6^ Moreover, some studies have revealed that the prolonged activation of plateau potentials leads to the sustained firing of lumbosacral motoneurons and the lower excitatory threshold, which leads to hyperexcitability of motor reflexes and muscle spasms.^7^ It is well known that L‐type voltage‐gated calcium channels (VGCC) are primarily responsible for the formation of plateau potentials, which were caused by the specific persistent inward currents (PICs) of spinal motoneurons.^6^, ^8^, ^9^ Nimodipine, an L−/T‐type VGCC blocker, can prevent spasticity in SCI rodents when administered beforehand^10^ and can promote the functional recovery in SCI rats.^11^ Also, the T‐type VGCC, which is thought to be mainly implicated in pain transmission and sensitization, involves the remodeling of neuronal and reflex excitability after SCI.^12^ Previous studies have reported that the expression of T and L VGCCs was changed after SCI, which might be associated with the functional alterations of neurons.^13^, ^14^ Nevertheless, the effect of T and L‐type VGCC blockers in SCI‐induced DSD has not been investigated yet. Meanwhile, 5‐HT, as a central neuromodulator, has been reported to regulate the plateau potential, thereby affecting the excitability of motoneurons.^15^ Hence, we hypothesized that 2,5‐dimethoxy‐4‐iodophenyl‐2‐aminopropane hydrochloride ameliorates DSD in SCI rats by modulating VGCC.

Therefore, our main purpose is to clarify the processes by which 5‐HT regulates specific VGCC to ameliorate DSD in rats with SCI, as well as to further investigate the modifications in VGCC subtypes within lumbosacral cord motoneurons.

## 2 MATERIALS AND METHODS

### 2.1 Animals

Female Sprague–Dawley rats weighing 250–350 g and aged 8 to 12 weeks were used. Animals were housed with free access to food and water under a 12‐by‐12 light–dark cycle. All experimental procedures were sanctioned by our Institutional Ethics Committee, and all procedures were performed in accordance with the guidelines established by the Animal Care and Use Committee of Shanghai Sixth People's Hospital and the National Research Council's Guide for the Care and Use of Laboratory Animals.

### 2.2 Animal model establishment and experimental design

Rats were randomly assigned to normal control group (control, *N* = 15) and spinal cord injury group (SCI, N = 15). The detailed methods of animal model establishment have been reported in our previous study,^16^ and briefly described as T10 level spinal cord transection performed in SCI rats. 4 to 8 weeks after SCI, or randomly in the control group, cystometrogram (CMG), simultaneous recording of CMG, and external urethral sphincter electromyography (EUS‐EMG) were performed in urethane‐anesthetized rats. Initially, only CMG was performed to explore the effects of L and T‐type calcium channel blockers on urinary function in both control and SCI rats (control, *N* = 7; SCI, *N* = 7). The intrathecal injection procedure during CMG is a sequential dosing regimen of 2,5‐dimethoxy‐4‐iodophenyl‐2‐aminopropane hydrochloride, T‐type VGCC blocker, and L‐type VGCC blocker with intervals of at least 30 min between administrations. Subsequently, based on the CMG findings, simultaneous recording of CMG and EUS‐EMG was conducted (control, *N* = 8; SCI, N = 8) with sequential intrathecal injections of 2,5‐dimethoxy‐4‐iodophenyl‐2‐aminopropane hydrochloride, L‐type VGCC blocker, and a combination of 2,5‐dimethoxy‐4‐iodophenyl‐2‐aminopropane hydrochloride and L‐type VGCC agonist. Following the functional studies, rats were euthanized with sodium pentobarbital, and spinal cord tissues were collected for immunofluorescence study.

### 2.3 Drugs

The following drugs were purchased from Sigma Aldrich (St. Louis, Missouri, USA): urethane (U2500), penicillin (PENNA), ibuprofen (I4883), 2,5‐dimethoxy‐4‐iodophenyl‐2‐aminopropane hydrochloride (D101, DOI, 5‐HT_2A/2C_ receptor agonist), (±)‐Bay K 8644 (L‐type voltage‐gated calcium channel agonist), nifedipine (N7364, L‐type voltage‐gated calcium channel blocker), rabbit anti‐CaV1.2 (1:200, C1241, CACNA1C) antibody, rabbit anti‐CaV1.3 (1:200, HPA020215, CACNA1D) antibody, rabbit anti‐CaV3.1 (1:200, SAB2104299, CACNA1G) antibody, mouse 5‐HT_2A_ receptor antibody (1:100, MABN1595). TTA‐P2 (HY‐10035, T‐type voltage‐gated calcium channel blocker) was purchased from MedChem Express (MCE, New Jersey, USA). Goat anti‐Choline Acetyltransferase (ChAT) antibody (1:200, ab254118) was purchased from Abcam (Massachusetts, USA). All secondary antibodies and normal donkey serum were purchased from YEASON (Shanghai, China). 2,5‐dimethoxy‐4‐iodophenyl‐2‐aminopropane hydrochloride, nifedipine, TTA‐P2, (±)‐Bay K 8644 were dissolved in 20% Dimethyl sulfoxide that did not change the contractile response of the detrusor and external urethral sphincter. All other drugs were freshly prepared by dissolving in distilled water.

### 2.4 CMG parameters before and after i.t injection of L and T‐type calcium channel blockers

The CMG was performed according to previous protocols.^4^ Rats were anesthetized with urethane (90 mg/kg, intraperitoneally). Before the CMG test, a polyethylene catheter (PE‐10; Smiths Medical Ltd, Ashford, Kent, UK) was inserted into the lumbosacral cord (L6–S1) for drug administration. The rats were allowed a 2‐h rest period following intrathecal catheterization. The urinary bladder was then accessed through a low midline abdominal incision. A PE‐50 catheter (Smiths Medical Ltd, Ashford, Kent, UK) was then carefully inserted into the bladder dome to measure intravesical pressure (IVP). The other end of the bladder catheter was connected to a pressure transducer and recorder (AD Instruments, Bella Vista, NSW, Australia). Continuous infusion of physiological saline at room temperature was maintained in the bladder, with infusion rates set at 0.1 mL/min for control rats and 0.2–0.6 mL/min for SCI rats. The higher infusion rate in SCI rats was necessary to ensure voiding contractions occurred within 10 min following drug administration. Once bladder contractions had reached a stable state lasting at least 30 min, injections were sequentially administered into the lumbosacral cord. The injections include 0.9% saline (10 μL), followed by 2,5‐dimethoxy‐4‐iodophenyl‐2‐aminopropane hydrochloride, TTA‐P2, and nifedipine. Using a microsyringe (Hamilton, Switzerland), each drug was injected every 30 min at a dose of 0.01 mg/kg in a 10 μL volume, as determined from prior research results.

### 2.5 Simultaneous recording of CMG and EUS‐EMG

Simultaneous recording of CMG and EUS‐EMG was conducted following the same intrathecal catheterization and CMG procedure outlined in section 2.4. The methodology for EUS‐EMG was as follows: Through an incision above the pubic bone, the EMG electrodes were placed in the sphincter position (one electrode on each side of the sphincter), with a third electrode placed on the abdominal skin for grounding. The EMG electrodes were connected to an amplifier and recorder with a sampling rate of 1000 Hz (NeuroStudio, Jiangsu Brain Medical Technology Co., Ltd., China).

### 2.6 Immunofluorescence study

Following the functional studies, rats were transcardially perfused with phosphate‐buffered saline (PBS, Serviobio, Wuhan, China). The L6‐S1 spinal cord was immersed in 4% paraformaldehyde for 2 h before being transferred into 30% sucrose for 72 h at 4°C. Spinal cord tissues were cut into 12 μm‐thick slices after embedding in optimal cutting temperature compound (Leica, Germany) (one section collected every 10 sections). Double‐labeled immunofluorescence of ChAT and VGCC subtypes, as well as ChAT and 5‐HT_2A_ receptors, was then performed. The primary antibodies used were goat anti‐ChAT antibody, mouse anti‐5‐HT_2A_ receptor, rabbit anti‐Ca_V_1.2 (CACNA1C) antibody, rabbit anti‐Ca_V_1.3 (CACNA1D) antibody, rabbit anti‐Ca_V_3.1 (CACNA1G) antibody.

### 2.7 The analysis of EUS‐EMG and CMG recordings

CMG parameters included: voiding volume (VV), residual volume (RV = Infusion volume—VV), basal pressure (the lowest bladder pressure observed during filling), threshold pressure for inducing voiding (the pressure recorded just before voiding), peak bladder pressure, and voiding efficiency (VE = VV/infusion volume × 100%), number of high frequency oscillations (HFOs). EUS‐EMG parameters included activity duration, bursting activity duration, and frequency of bursting activity. The EMG data were filtered and analyzed using MATLAB (R2022b, MathWorks, Boston, MA, USA).

### 2.8 The analysis of the immunofluorescence study

After immunofluorescence staining, microphotographs were acquired using a slide scanner (camera: Pannoramic MIDI, 3DHISTECH, Hungary; objective: Plan‐Apochromat 20×/0.8). Fixed‐sized areas were selected for L6‐S1 spinal ventral cell counting. Within these areas, neurons were distinguished primarily by the size of their cells and their nuclei. Only neurons with nuclei stained with DAPI were counted to prevent double counting. The number of positive neurons was identified and analyzed using image analysis software (Image J, NIH, USA).

### 2.9 Statistical analysis

All statistical analyses were conducted using Prism 9 (GraphPad Software, Boston, MA, USA). The normal distribution of data was tested with Kolmogorov–Smirnov test and Shapiro–Wilk test. Unpaired *t*‐tests and repeated‐measures analysis of variance (ANOVA) were used when the assumptions of normal distribution and homogeneity of variances were met. Nonparametric tests were used when the data did not meet these assumptions. All statistical tests were two‐tailed. Data are primarily represented as histograms with bars and data points, and bars represent mean ± SEM unless specifically stated. The threshold level for *α* was defined as *p* < 0.05 and corrected for multiple comparisons. Significance levels were indicated as n.s. (not significant, *p* > 0.05), *p < 0.05, **p < 0.01, ***p < 0.001.

## 3 RESULTS

### 3.1 The effect of L and T type VGCC blockers on the CMG parameters in control and SCI rats

**FIGURE 1:**
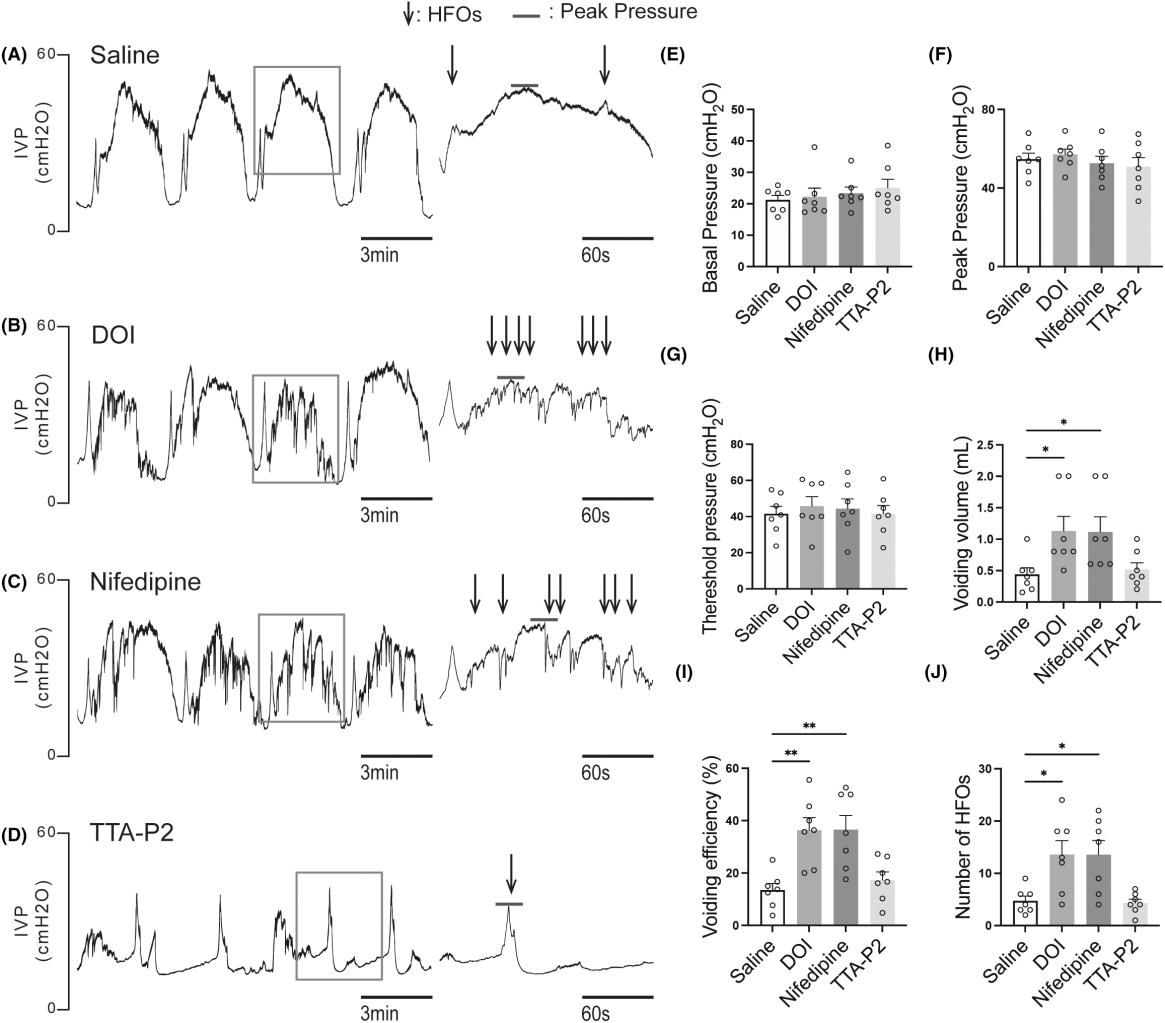
The effect of DOI, nifedipine, and TTA‐P2 on CMG in SCI rats. (A–D) Representative time course images of CMG after i.t injection of saline (control), 2,5‐dimethoxy‐4‐iodophenyl‐2‐aminopropane hydrochloride, nifedipine, TTA‐P2, with the right side shows selected magnified segments of 180 s (indicated by the transparent box). (E–J): CMG parameters of SCI rats (*N* = 7). 2,5‐dimethoxy‐4‐iodophenyl‐2‐aminopropane hydrochloride and nifedipine significantly increased voiding volume and enhanced voiding efficiency in SCI rats, whereas TTA‐P2 had no discernible effect (E–I). Furthermore, both 2,5‐dimethoxy‐4‐iodophenyl‐2‐aminopropane hydrochloride and nifedipine increased the number of high‐frequency oscillations (HFOs) in SCI rats (A–D, J), suggesting that they enhance urethral function. The normality of the data were assessed using the Kolmogorov–Smirnov test and Shapiro–Wilk test and the results indicated that the data followed a normal distribution (*p* > 0.05). Each dot represents one rat. Mean ± SEM, one‐way ANOVA, Dunnett's multiple comparisons test with homogeneity of variance. *p < 0.05; **p < 0.01.

CMG parameters were not significantly affected by 2,5‐dimethoxy‐4‐iodophenyl‐2‐aminopropane hydrochloride, the L‐type VGCC blocker nifedipine, or the T‐type VGCC blocker TTA‐P2 in control rats. However, 2,5‐dimethoxy‐4‐iodophenyl‐2‐aminopropane hydrochloride and nifedipine significantly increased voiding volume and enhanced voiding efficiency in rats with SCI, whereas TTA‐P2 had no discernible effect (Figure 1E–I). Furthermore, both 2,5‐dimethoxy‐4‐iodophenyl‐2‐aminopropane hydrochloride and nifedipine increased the number of HFOs in SCI rats (Figure 1A–D,J), suggesting that they enhance urethral function.

### 3.2 The changes of CMG and EUS‐EMG parameters in SCI rats compared to control rats

**FIGURE 2:**
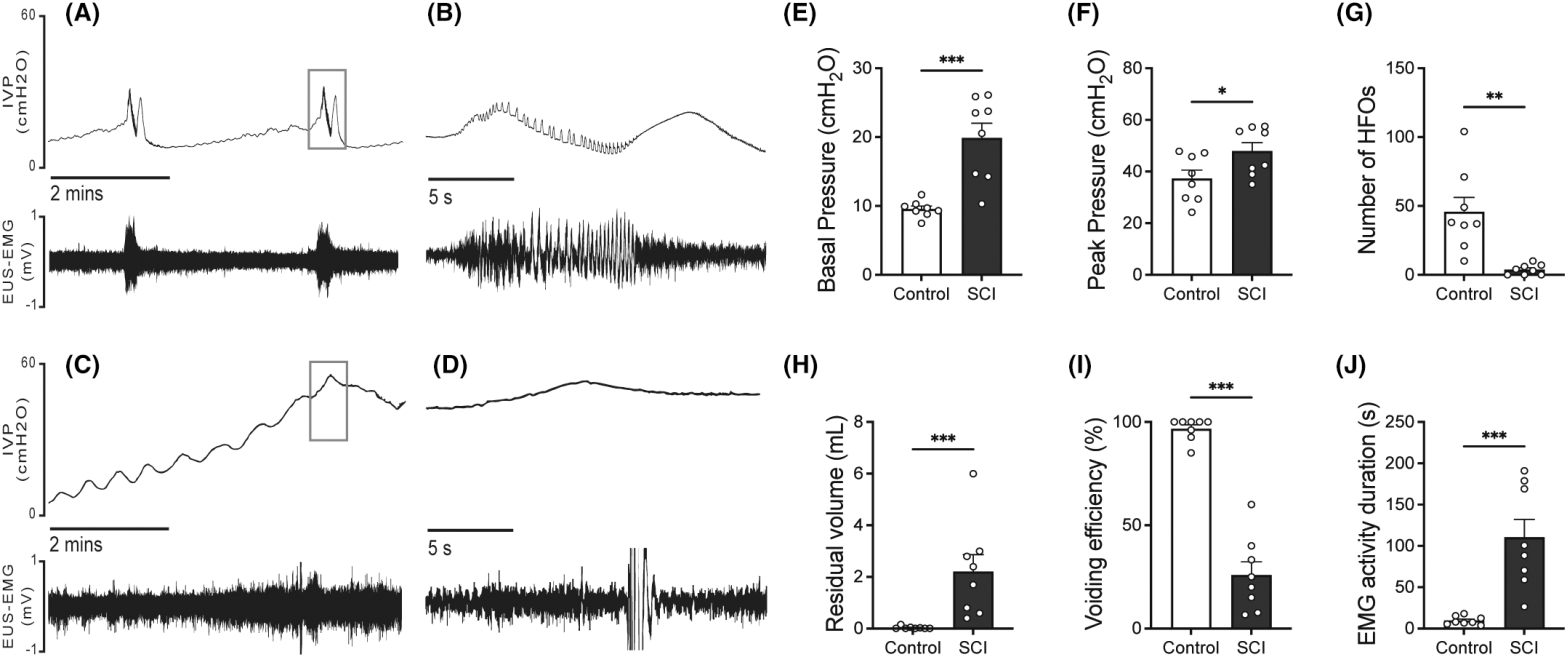
Changes of CMG and EUS‐EMG parameters in SCI rats compared to control rats. (A) and (B) Representative images of CMG and EUS‐EMG in control rats, the right side shows selected magnified segments of 20 s (indicated by the transparent box). (C) and (D) Representative images of CMG and EUS‐EMG in SCI rats, the right side shows selected magnified segments of 20 s (indicated by the transparent box). (E–J) CMG and EUS‐EMG parameters of control (*N* = 8) and SCI rats (N = 8). SCI rats exhibit prolonged voiding contractions with few HFOs (C, D), compared to the single, brief voiding contraction with regular HFOs in control rats (A, B). Basal bladder pressure, peak pressure, residual urine volume, HFOs, and voiding efficiency show significant differences between SCI and control rats (E–J). The normality of the data was assessed using the Kolmogorov–Smirnov test and Shapiro–Wilk test and the results indicated that the data in E, F, G, J followed a normal distribution (*p* > 0.05). Student's *t*‐test was used for the analyzation. Data in panels (H) and (I) did not exhibit a normal distribution and were analyzed with Mann–Whitney test. Each dot represents one rat. Mean ± SEM. *p < 0.05; **p < 0.01; ***p < 0.001.

Based on the preceding CMG results, we aimed to further elucidate how 2,5‐dimethoxy‐4‐iodophenyl‐2‐aminopropane hydrochloride and L‐type calcium channel modulation impacted urethral function by simultaneously recording of CMG and EUS‐EMG. After opening the abdomen to perform CMG bladder catheterization, several evident differences were observed between the bladders of SCI rats and those of control. In particular, SCI rats exhibited distention and hypertrophy of the bladders in comparison to control. Additionally, control rats exhibited a single, brief voiding contraction with regular HFOs during voiding in the CMG (Figure 2A,B). On the contrary, SCI rats revealed considerably extended voiding contractions with little or absent HFOs (Figure 2C,D). The basal bladder pressure during the filling phase and peak pressure of voiding contractions were notably higher in SCI rats (Figure 2E,F). Additionally, residual urine volume was significantly greater in SCI rats compared to control rats, while the number of HFOs and voiding efficiency were lower in SCI rats compared to control (Figure 2G–J).

In control rats, EMG activity of the EUS exhibited a regular increase with each sub‐bladder contraction. This activity was characterized by bursting events corresponding to the HFOs in CMG, featuring a frequency of 5.274 ± 0.4495 Hz and lasting 9.717 ± 1.787 s. Conversely, SCI rats displayed persistent irregular increases in EUS‐EMG activity, which were significantly prolonged (110.5 ± 21.73 s) and incorporated irregular tonic activity (Figure 2, C and D).

### 3.3 5‐HT_2A_ _/2C_ receptor agonist 2,5‐dimethoxy‐4‐iodophenyl‐2‐aminopropane hydrochloride may improve DSD by inhibiting L‐type voltage‐gated calcium channels in SCI rats

**FIGURE 3:**
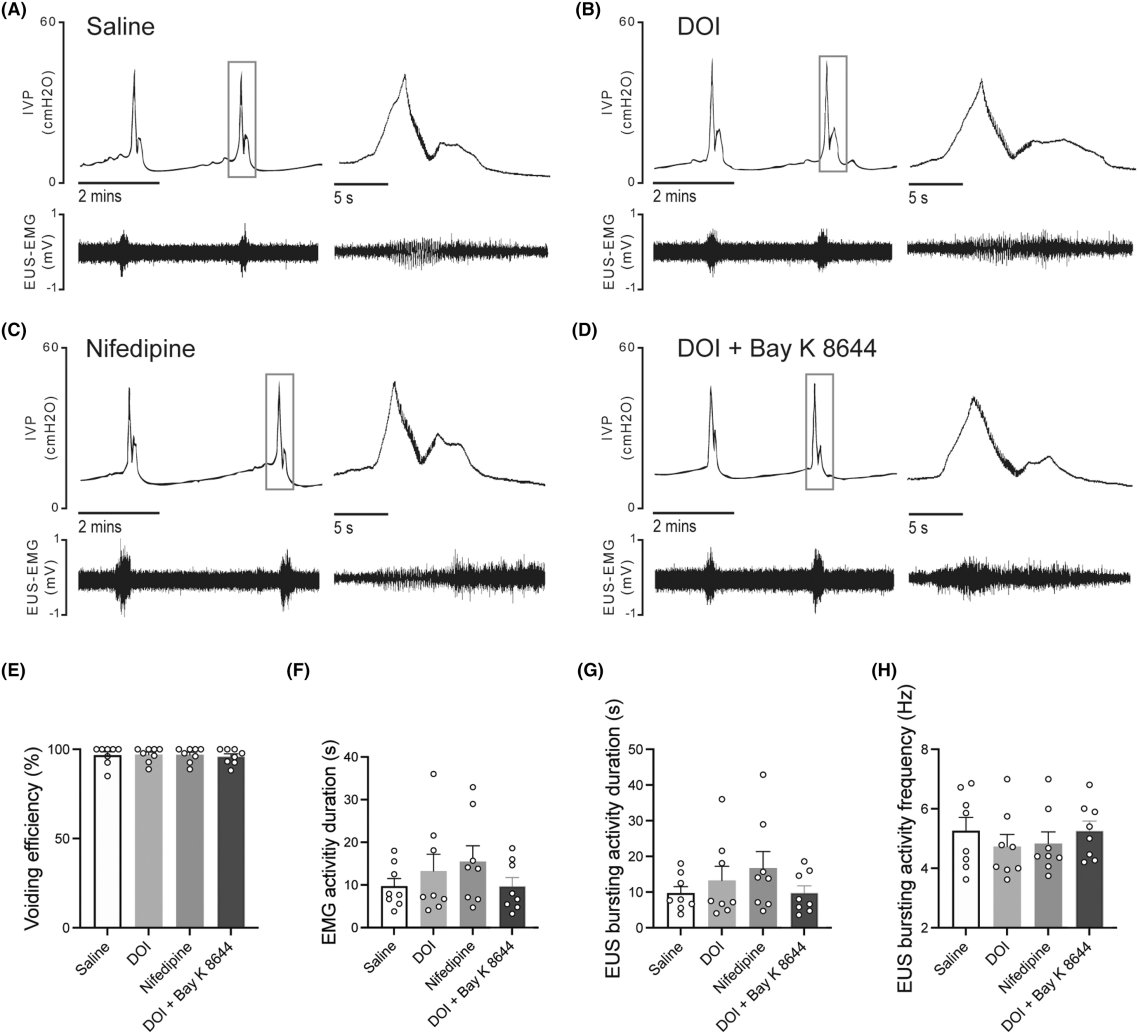
The effect of DOI, nifedipine, combined DOI and Bay K 8644 on simultaneous CMG and EUS‐EMG in control rats. (A) to (D) Representative images of CMG and EUS‐EMG after i.t injection of saline (control), 2,5‐dimethoxy‐4‐iodophenyl‐2‐aminopropane hydrochloride, nifedipine, combined 2,5‐dimethoxy‐4‐iodophenyl‐2‐aminopropane hydrochloride and Bay K8644. (E) to (H) CMG and EUS‐EMG parameters of control rats (*N* = 8), and they weren't affected by i.t administration of these drugs. The normality of the data was assessed using the Kolmogorov–Smirnov test and Shapiro–Wilk test and the results indicated that the data followed a normal distribution (*p* > 0.05). Each dot represents one rat. Mean ± SEM, one‐way ANOVA, Dunnett's multiple comparisons test with homogeneity of variance. None of the analysis is significant.

**FIGURE 4:**
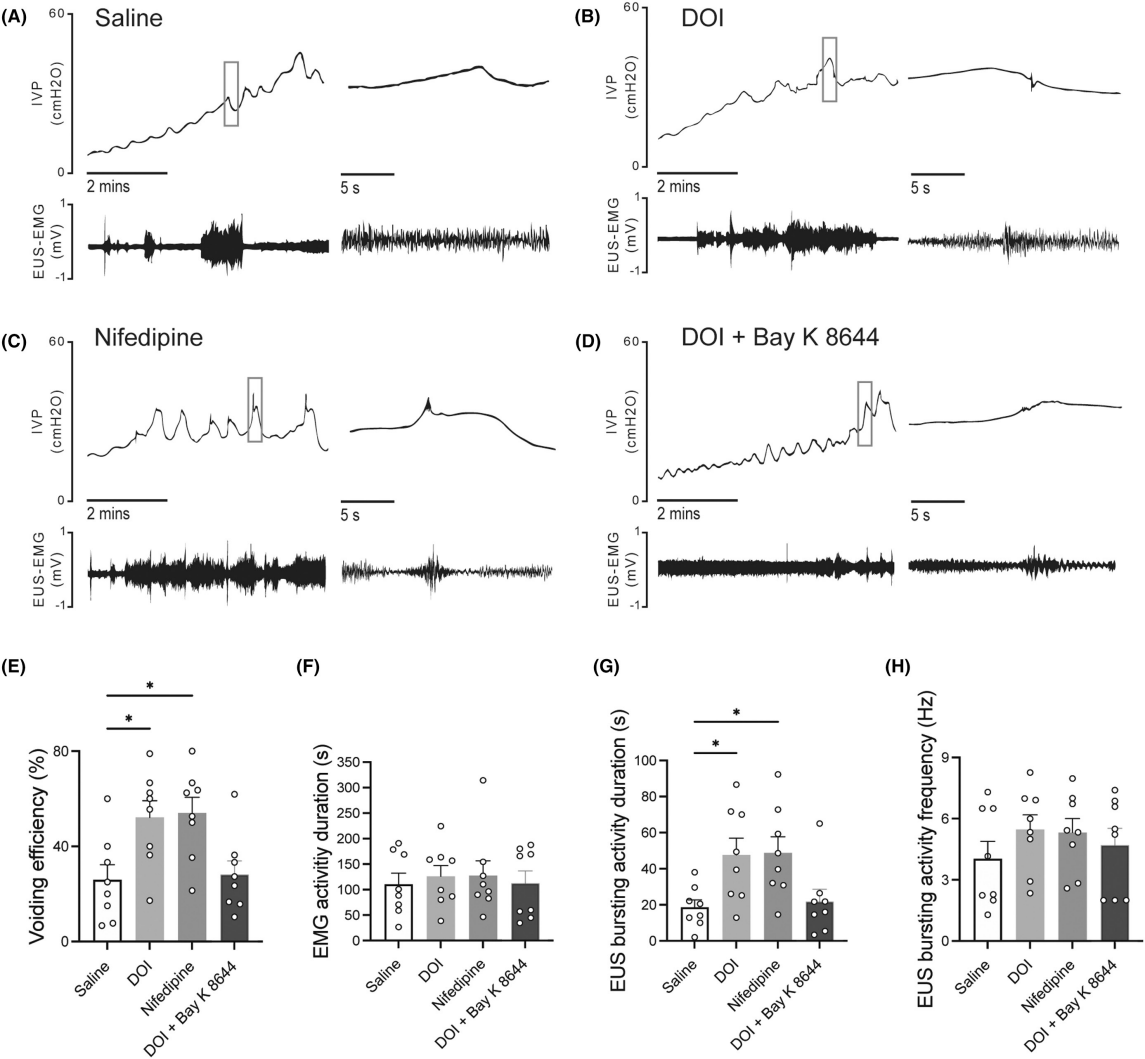
The effect of DOI, nifedipine, combined DOI and Bay K 8644on simultaneous CMG and EUS‐EMG in SCI rats. (A–D) Representative images of CMG and EUS‐EMG after i.t injection of saline (control), 2,5‐dimethoxy‐4‐iodophenyl‐2‐aminopropane hydrochloride, nifedipine, combined 2,5‐dimethoxy‐4‐iodophenyl‐2‐aminopropane hydrochloride and Bay K8644. (E) to (H) CMG and EUS‐EMG parameters of SCI rats (N = 8). 2,5‐dimethoxy‐4‐iodophenyl‐2‐aminopropane hydrochloride and nifedipine significantly increased voiding efficiency in SCI rats (E). Intrathecal administration of nifedipine alone mimicked the effects of 2,5‐dimethoxy‐4‐iodophenyl‐2‐aminopropane hydrochloride in improving voiding efficiency and prolonging bursting activity duration (B, C, E, and G). However, the combination administration of 2,5‐dimethoxy‐4‐iodophenyl‐2‐aminopropane hydrochloride and (±)‐Bay K 8644 eliminated the efficacy of 2,5‐dimethoxy‐4‐iodophenyl‐2‐aminopropane hydrochloride in improving SCI‐induced detrusor‐sphincter dyssynergia (D, E, and G). The normality of the data were assessed using the Kolmogorov–Smirnov test and Shapiro–Wilk test and the results indicated that the data followed a normal distribution (*p* > 0.05). Each dot represents one rat. Mean ± SEM, one‐way ANOVA, Dunnett's multiple comparisons test with homogeneity of variance. *p < 0.05.

Intrathecal administration of 2,5‐dimethoxy‐4‐iodophenyl‐2‐aminopropane hydrochloride and nifedipine did not evidently affect the EUS‐EMG of control rats evidently (Figure 3A–H). However, they significantly increased VV and improved voiding efficiency in SCI rats (Figure 4E). The EMG results have shown that SCI rats exhibited prolonged bursting activity duration (Figure 4A), while no significant difference was observed in the frequency of bursting activity (Figure 4H). As is similar to 2,5‐dimethoxy‐4‐iodophenyl‐2‐aminopropane hydrochloride, i.t. administration of nifedipine could improve voiding efficiency and prolong the bursting activity duration in SCI rats (Figure 4B,C,E,G). After the combination administration of 2,5‐dimethoxy‐4‐iodophenyl‐2‐aminopropane hydrochloride and L‐type VGCC agonist (±)‐Bay K 8644, the effect of 2,5‐dimethoxy‐4‐iodophenyl‐2‐aminopropane hydrochloride in improving SCI‐induced DSD was eliminated in SCI rats (Figure 4D).

### 3.4 The expressions of 5‐HT_2A_ receptors and VGCC subtypes in lumbosacral cord motoneurons in SCI rats

**FIGURE 5:**
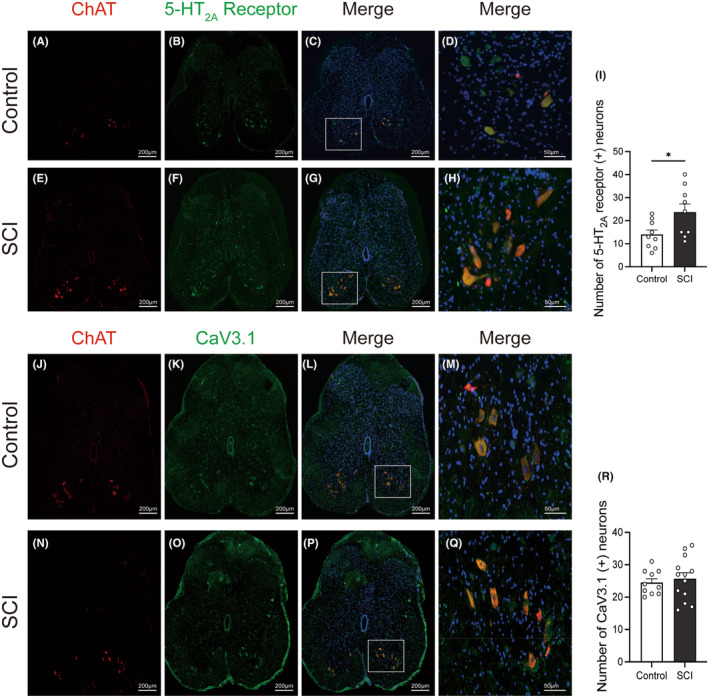
Representative images of immunofluorescence staining illustrating the expression patterns of 5‐HT2A receptors and T‐type VGCC (CaV3.1) in the lumbosacral cord. Motoneuronal localization is confirmed through co‐staining with ChAT, a motoneuron marker (A, E, J, N). Following SCI, the expression of Cav 3.1 (T‐type VGCC) remains relatively unchanged (K, O, R), while a significant increase is observed in the population of motoneurons expressing 5‐HT2A receptors (B, F, I). Merged photos of ChAT and immunofluorescent signals for Cav3.1, 5‐HT2A, (C, G, L, P) further illustrate the colocalization within motoneurons, providing a comprehensive view of the protein distribution in the lumbosacral cord. Magnified views of the boxed regions (D, H, M, Q) and corresponding histograms (I, R) highlight the specific colocalization patterns and compare immuno‐positive neurons in the anterior horn between control rats and SCI rats. The normality of the data was assessed using the Kolmogorov–Smirnov test and Shapiro–Wilk test and the results indicated that the data followed a normal distribution (*p* > 0.05). Each dot represents one spinal slice from each rat. Mean ± SEM, Student's *t*‐test. *p < 0.05. Scale bar: 50 μm for (D, H, M, Q), 200 μm for the rest.

**FIGURE 6:**
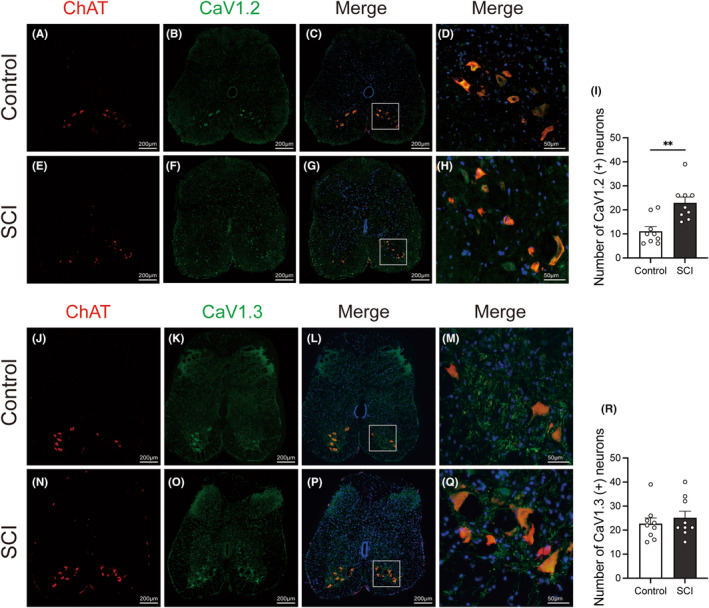
Representative images of immunofluorescence staining illustrating the distribution of L‐type VGCC subtypes (Cav1.2 and Cav1.3) in the lumbosacral cord. Co‐staining with ChAT, a motoneuron marker (A, E, J, N), confirms predominant expression within neurons. Following SCI, the count of neurons positive for Cav1.2 (L‐type VGCC) significantly increases (B, F, I), whereas no significant change is observed in Cav1.3‐positive neurons (K, O, R). Merged photos of ChAT and immunofluorescent signals for Cav1.2 and Cav1.3 (C, G, L, P) provide a visual representation of the colocalization within motoneurons, offering insight into the differential regulation of L‐type VGCC subtypes after SCI. Magnified views of the boxed regions (D, H, M, Q) and corresponding histograms (I, R) highlight the specific colocalization patterns and compare immuno‐positive neurons in the anterior horn between control rats and SCI rats. The normality of the data was assessed using the Kolmogorov–Smirnov test and Shapiro–Wilk test and the results indicated that the data followed a normal distribution (*p* > 0.05). Each dot represents one spinal slice from each rat. Mean ± SEM is shown, and statistical analysis was performed using Student's *t‐*test. *p < 0.05. Scale bar: 50 μm for (D, H, M, Q), 200 μm for the rest.

The expressions of the 5‐HT_2A_ receptors, L‐type VGCC (Cav1.2 and 1.3), and T‐type VGCC were examined by immunofluorescence staining. These proteins were predominantly localized within neurons rather than glial cells. Through co‐staining with ChAT, a motoneuron marker (A, E J, N of Figure 5 and Figure 6), we found that these proteins were expressed in motoneurons located in the anterior horn of the lumbosacral cord (D, H M, Q of Figures 5 and 6). After SCI, the expression of Cav 3.1(labeling T‐type VGCC) in lumbosacral cord anterior horn remained relatively unchanged (Figure 5K,O,R). Moreover, the population of neurons expressing 5‐HT_2A_ receptor (Figure 5B,F,I) and Cav1.2 (labeling L‐type VGCC) (Figure 6B,F,I) were significantly increased in the anterior horn of the lumbosacral cord. In contrast, the number of neurons positive for Cav1.3 (labeled for L‐type VGCC) did not show a significant change in SCI rats (Figure 6K,O,R).

## 4 DISCUSSION

The current study aimed to examine the involvement of L‐ and T‐type VGCCs in the improvement of DSD following administration of 2,5‐dimethoxy‐4‐iodophenyl‐2‐aminopropane hydrochloride in SCI rats. Moreover, we evaluated the changes in the 5‐HT_2A_ receptor, L‐ and T‐type VGCCs in spinal motoneurons after SCI. In this study, some results could be concluded as follows: First, i.t. administration of 2,5‐dimethoxy‐4‐iodophenyl‐2‐aminopropane hydrochloride primarily improves DSD through the enhancement of EUS bursting activity. Second, L‐type VGCC blocker nifedipine rather than T‐type blocker TTA‐P2 could improve SCI‐induced DSD in a manner similar to 2,5‐dimethoxy‐4‐iodophenyl‐2‐aminopropane hydrochloride. Thirdly, the effects of 2,5‐dimethoxy‐4‐iodophenyl‐2‐aminopropane hydrochloride in SCI‐induced DSD were eliminated when combined administration of 2,5‐dimethoxy‐4‐iodophenyl‐2‐aminopropane hydrochloride and L‐type VGCC agonist (±)‐Bay k8644. These functional results suggest that 2,5‐dimethoxy‐4‐iodophenyl‐2‐aminopropane hydrochloride may improve DSD by inhibiting L‐type VGCC in SCI rats.

Rats, unlike humans, exhibit unique EUS activity during voiding that is crucial for bladder emptying, as revealed by CMG and EUS‐EMG, aligning with earlier studies. Normal voiding in rats involves four phases: an increase in intravesical pressure (IVP) aligned with EUS‐EMG tonic activity, followed by the appearance of HFO on CMG, a subsequent decrease in IVP with EUS‐EMG bursting activity, a rebounding in IVP with EUS‐EMG returning to tonic activity, and a dramatic IVP fall indicating bladder emptying.^1^, ^17^ These results were significantly changed in SCI rats. As is shown in Figure 2. SCI rats lack these phases and show reduced or absent bursting activity, prolonged voiding time/EMG duration, and elevated IVP, indicating significant DSD symptoms. DSD can be ascribed to the loss of upper neuronal guarding reflexes and local spinal cord pathophysiological alterations after SCI, which cause the inappropriate excitation of Onuf's nucleus (motoneurons of lumbosacral cord innervating EUS).^18^

Spinal 5‐HT originates from the raphe nucleus in the caudal brainstem.^19^ Consequently, following a spinal cord transection, the downstream 5‐HT level drops dramatically. Thus, the upregulation of the 5‐HT_2A_ receptor after SCI may be compensatory. Although the firing of spinal neurons is predominantly mediated by glutamate and GABA, neuromodulatory neurotransmitters such as 5‐HT play an important regulatory role in this process. Therefore, after SCI, the diminished or absent 5‐HT input in the spinal cord below the site of injury may lead to various pathological conditions.^20^ 2,5‐dimethoxy‐4‐iodophenyl‐2‐aminopropane hydrochloride can selectively activate 5‐HT_2A/C_ receptors of the central nervous systems.^21^ After i.t. injection of 2,5‐dimethoxy‐4‐iodophenyl‐2‐aminopropane hydrochloride, significant effects were observed in SCI rats. First, the voiding volume was significantly increased, while the residual volume was significantly decreased, leading to a significant increase in voiding efficiency. These results emphasize the potential efficacy of 2,5‐dimethoxy‐4‐iodophenyl‐2‐aminopropane hydrochloride in ameliorating voiding dysfunction after SCI, which aligns with the results of our earlier studies.^4^ Notably, we also observed a significant increase in the number of HFOs. This phenomenon was further validated in EUS‐EMG, showing a prolonged duration of EUS bursting activity. This suggests that the application of 2,5‐dimethoxy‐4‐iodophenyl‐2‐aminopropane hydrochloride improved DSD in SCI rats.

Interestingly, comparable effects were observed after the administration of nifedipine instead of TTA‐P2, which included a prolongation of voiding time, and an increase in voiding efficiency. These observations offer indications that the DSD after SCI may be associated with the overactivation of calcium channels. Nifedipine, a specific L‐type VGCC antagonist, functions by inhibiting the entry of calcium ions into cells.^22^ There are dual possible explanations. One is that the i.t. administration of nifedipine directly blocked L‐type calcium channels and lowered the excitability in spinal cord, thus decreasing the tonic activity of EUS. The other explanation could be attributed to the involvement of plateau potentials of motoneurons. The spinal cord motoneurons have a specific plateau potential. They are usually of long duration and can prolong and intensify the firing state, thereby influencing muscle contraction and maintaining specific movement patterns. Plateau potentials are usually the result of PICs. PICs are induced mainly through sodium and calcium channels.^8^ The relative importance of sodium and calcium channels in the formation of the PICs varies between spinal cord segments. Since nifedipine, rather than TTX, can block PICs, L‐type VGCC is considered to be mainly involved. SCI leads to a temporary loss of plateau potentials in the early stage of trauma, partly explaining the phenomenon of spinal shock period. However, as rehabilitation proceeds, the plateau potential gradually recovers but it lacks normal regulation by upstream neurotransmitters, leading to an overactive state. After SCI, there are often hyperreflexia and tonic contractions of muscles, which may be due to prolonged reflex responses. Furthermore, the firing was accelerated and irregular in chronic spinal rat motoneurons.^23^ Some researchers use nimodipine, also an L‐type VGCC blocker, to treat and prevent the spasticity of tails after SCI in mice. They found that nimodipine completely prevents the development of spasticity and eliminates abnormal muscle activity even after treatment was stopped. They also stated that Cav1.3 induced the effect.^10^ The L‐type VGCC has three subtypes, namely, Cav1.1 to 1.3. In the spinal cord, mainly Cav1.2 and Cav1.3 are expressed. According to our results, both Cav1.2 and Cav1.3 are widely expressed in spinal neurons and fibers including Onuf's nucleus. After SCI, the number of Cav1.2 positive neurons has elevated significantly while that of Cav1.3 has no obvious changes. According to the study of R. ANELLI et al., the expression of Cav1.2 was increased and Cav1.3 remained unchanged after SCI.^13^ These results indicate that Cav1.2 and Cav1.3 may have different roles during the pathophysiology of SCI. Based on our results, nifedipine may exert its effect in improving DSD by potentially inhibiting the Cav1.2 subunit of L‐type VGCC given the upregulation of Cav1.2 in lumbosacral anterior horn. Alternatively, another possible explanation is that, although the expression level of Cav1.3 remains unchanged, its activity might be altered following SCI. This requires subunit‐specific L‐type VGCC blockers and further electrophysiological experiments to validate. Since T‐type calcium channel blockers did not yield significant functional results in functionality, we only evaluated the subtype Cav3.1 in immunofluorescence. The T‐type calcium channel, which is generally considered to be primarily associated with sensation, is also widely expressed in neurons as well as nerve fibers in the lumbosacral cord and the expression level remained unchanged after SCI according to our immunofluorescence results.

Additionally, we found that the effect of 2,5‐dimethoxy‐4‐iodophenyl‐2‐aminopropane hydrochloride on the EUS vanished after combined administration of 2,5‐dimethoxy‐4‐iodophenyl‐2‐aminopropane hydrochloride and (±)‐Bay k8644. Previous studies have shown that monoamine neurotransmitters such as 5‐HT can modulate plateau potentials.^24^, ^25^ Therefore, considering the above discussion, we hypothesized that 2,5‐dimethoxy‐4‐iodophenyl‐2‐aminopropane hydrochloride may improve the irregular state of motoneuron plateau potentials, and thus improve DSD by inhibiting the activity of L‐type calcium channels in spinal cord motoneurons. These findings provide additional evidence to substantiate the theory that irregular urinary function following SCI is closely related to the degree of calcium channel activation. Excessive activation of calcium channels can result in DSD, thereby impacting the urinary process. Therefore, the application of L‐type voltage‐gated calcium channel antagonists like nifedipine holds the potential to rectify this anomaly, offering prospects for enhancing the urinary function of individuals with SCI.

There are some limitations in our study. First, functional experiments under anesthesia: We used anesthesia to manipulate the animals for this study. The physiological states can be different when awake. Anesthesia may alter neuron and muscle activity and physiological parameters. Thus, anesthesia may influence our results and not fully reflect the physiological state in the awake state. Second, the lack of electrophysiology experiments: this study examined 5‐HT, calcium channels, and DSD using functional experiments and immunofluorescence. Functionality and immunofluorescence reveal these mechanisms, but electrophysiology experiments are needed to confirm our findings. Electrophysiological experiments can shed light on neuronal activity and ion channel function, thereby improving our understanding of the mechanisms. Therefore, additional studies are needed to overcome these limitations. Future research could include awake experiments to better reflect the physiological situation and electrophysiological studies to confirm our hypotheses and conclusions. This will enhance understanding of SCI‐related voiding dysfunction mechanisms and support treatment development.

## 5 CONCLUSIONS

5‐HT_2A/2C_ agonist 2,5‐dimethoxy‐4‐iodophenyl‐2‐aminopropane hydrochloride may improve SCI‐induced DSD by inhibiting the L‐type voltage‐gated calcium channel in lumbosacral cord motoneurons. These findings are helpful to clarify the pharmacological effects of 2,5‐dimethoxy‐4‐iodophenyl‐2‐aminopropane hydrochloride and reveal a new potential therapeutic target for SCI‐induced DSD in clinical practices.

## AUTHOR CONTRIBUTIONS

Rong Lv: Formal Analysis, methodology, writing—original draft; Mingzhuo Li: Methodology; Xun Chen: Methodology; Shengtian Li: supervision, writing—review & editing; Nailong Cao: Conceptualization, writing—review & editing; Baojun Gu: Conceptualization, writing—review & editing, supervision.

## FUNDING INFORMATION

This research was funded by the Shanghai Sailing Program [grant number: 21YF1434400]; National Natural Science Foundation of China [grant number: 82070786]; The Youth cultivation project of Shanghai Sixth People's Hospital Affiliated to Shanghai Jiao Tong University School of Medicine [grant number: ynqn202112].

## CONFLICT OF INTEREST STATEMENT

The authors declare no conflicts of interest.

## Data Availability

The data that support the findings of this study are available from the corresponding author upon reasonable request.
